# Frequency of recurrent stroke in Burkina Faso: an observational hospital based study of 6 months

**DOI:** 10.11604/pamj.2021.40.108.23098

**Published:** 2021-10-19

**Authors:** Alfred Anselme Dabilgou, Alassane Dravé, Julie Marie Adeline Kyelem, Robert Zoungrana, Christian Napon, Athanase Millogo, Jean Kaboré

**Affiliations:** 1 Department of Neurology, University Hospital Yalgado Ouedraogo, Ouagadougou, Burkina Faso,; 2 Department of Neurology, Regional University Hospital of Ouahigouya, Ouahigouya, Burkina Faso,; 3 Department of Neurology, University Hospital of Bogodogo, Ouagadougou, Burkina Faso,; 4 Department of Neurology, University Hospital Teaching Hospital Souro Sanon, Bobo Dioulasso, Burkina Faso

**Keywords:** Frequency, stroke recurrence, vascular risk factors, Burkina Faso

## Abstract

**Introduction:**

studies on stroke recurrence are rare in sub-Sahara Africa. The aim to this study is to determine the prevalence and risk factors for recurrent stroke in two University Teaching Hospital in Burkina Faso.

**Methods:**

this prospective cross-sectional study was carried on 266 stroke patients admitted in two hospitals in the city of Ouagadougou from September 1, 2017 to February 28, 2018. Patients with stroke recurrence (ischemic or hemorrhagic) were included.

**Results:**

of 266 acute stroke patients included, 44 (16.4%) had recurrent stroke. The mean age of patients was 66.5 ± 11.49 years with male predominance. Hypertension was the most vascular risk factors (81.8%). Previous stroke was ischemic in 61.4%, hemorrhagic in 22.7% and unknown in 15.9% of cases. Poor compliance (< 60%) was determined in patients taking antiagregant (43.6%) and statins (50%). At admission, the most neurological disorders was motor deficit (100%), aphasia (84.1%), and deglutition disorders (15.9%). CT scan showed ischemic in 82% and hemorrhagic stroke in 18% of cases. With the analysis of second stroke, recurrent stroke after intracerebral hemorrhage was hemorrhagic in 77.8% and ischemic in 22.2%. Recurrent stroke after ischemic stroke was ischemic in 100%.

**Conclusion:**

stroke recurrence is common in our context. Hypertension was the most common vascular risk factor in recurrent stroke. Poor compliance was determined in patients taking antiagregant agents and statins in previous stroke.

## Introduction

Stroke remains the second leading cause of death worldwide, with 5.5 million deaths attributed to this cause in 2016 [1]. Recurrent stroke is a major contributor to disability and mortality in patients with stroke [2]. Several studies in western countries have shown that the recurrence risk was 11.2% within 12 months; 15% after 2 year and 9.5% within 5 years [3-5]. Africa appears to have the highest incidence, prevalence and case fatality of stroke [6-10]. There were few studies on recurrent stroke in sub-Saharan Africa, particularly in Cameroon, Nigeria and Ethiopia [11-13]. In Burkina Faso, there was no data concerning the frequency of recurrent stroke. The aims of this study was to determine the frequency of recurrent stroke in a tertiary hospital in order to improve the management and secondary prevention of stroke.

## Methods

**Study profile**: this study was conducted on stroke patients admitted to Yalgado Ouedraogo University Hospital Teaching Hospital and Bogodogo University Teaching Hospital during September 1, 2017 to February 28, 2018 (6 months). We included consecutive patients with recurrent ischemic or hemorrhagic stroke. Patients without consent and patients who had no information on their previous stroke record were excluded.

**Data collection and analysis**: we had collected the data on individual cards established for this purpose, and the information was collected from the patients, their accompanying persons and using the patient's medical file. All the patients had performed brain computed tomography (CT) and cervical ultrasound. Some investigations were done in selected cases (hemogram, electrolytes, blood glucose, cerebral magnetic resonance imaging (MRI), and lipid profile). The following variables were selected. Socio-demographic factors included age, gender, place of residence, occupation and educational level. Vascular risk factors included history of transient ischemic attack (TIA), ischemic heart disease, atrial fibrillation (AF), hypertension, alcohol, physical inactivity, obesity, diabetes mellitus and smoking. Clinical factors included (restroke onset, neurological deficit at admission, severity of restroke, stroke subtypes, and causes of restroke). The data will be processed using the Word and Excel 2016 software. Data entry and analysis will be done by Epidata software and Epi-info version 3.1.

**Ethical and deontological considerations**: prior to inclusion, informed consent from each patient or their relatives was obtained. We respected the confidentiality of the information given to us. The study was approved by the local ethical committee.

**Definitions**: stroke recurrence was defined as a new neurological deficit, including ischemic or hemorrhagic stroke, which occurs any time after the index stroke [14-16]. Stroke severity was defined as the presence of neurological deficit after last previous stroke. Case severity variables included motor deficit, urinary incontinence, and symptoms of depression. Vascular risk factors included arterial hypertension defined as previous medical treatment with antihypertensive or detected persistent blood pressure >140/90 mmHg, atrial fibrillation as evidenced by electrocardiography (ECG) or 24-hour Holter monitoring, smoking defined as current cigarette smoking during previous stroke. Alcohol consumption was defined as current drinking during previous stroke. Diabetes mellitus (previous medical treatment with anti-diabetic or detected fasting plasma glucose level of = 6.1 mmol/L or with symptoms of diabetes plus random plasma blood sugar of = 11.1 mmol/L or HbA1C (glycosylated hemoglobin) >6.5%), hyperlipidemia (previous medical treatment with anti-hyperlipidemia or detected total cholesterol =240 mg/dl or low-density lipoprotein (LDL) =160 mg/dl) and obesity (body mass index =30) were also regarded as possible predictors of stroke recurrence. Multivariable logistic regression analysis was employed to compare the factors that were possible predictors of recurrence amongst these patients with ischemic stroke. Data was analyzed using Epi Info.

## Results

**Frequency**: during study period, 266 stroke patients were included in this study. Forty-four (16.5%) patients were hospitalized due to recurrent stroke. Of them, 37 (13.9 %) were admitted for second stroke and 7(2.6%) for third stroke. There were 51 episodes of retroske, including 39 (76.5%) ischemic stroke, 9 (17.6%) hemorrhagic stroke and 3 (5.9%) unknown nature. Each patient had 1.15 restroke per patient. The Table 1 gives the frequency of overall stroke.

**Table 1:** characteristics of overall stroke

Characteristics	Value
Number of admitted patients	266
Ischemic	177(66.5%)
Hemorrhagic	89(33.5%)
Restroke	44(16.5%)
2times	37(13.9%)
3 times	7(2.6)
Lifetime number of stroke episodes of the 44 restroke patients	95
Ischemic stroke	66(69.5%)
Hemorrhagic stroke	18(18.9%)
Unknown nature	11(11.6%)

**Sociodemographic characteristics**: the mean age of the patients with stroke recurrence was 66.5 ± 11.49 years (ranges: 36-86 years). Older patients (≥ 60 years) accounted 72.7% (n=32). Young patients (≤ 45 years) accounted for 2.3% (n=1). This study included 32 (72.7%) males and 12 (27.3%) women. The mean age of men was 68.5±11.93 years (ranges: 36-86 years) and those of women was 57.5±8.97 years (ranges 49-74 years). There was no significant difference between age of men and women (p = 0.56). The mean age of ischemic stroke patients was 68.50±11.3 years and 73.8±8.5 years in recurrent hemorrhagic stroke. Table 2 gives the sociodemographic characteristics of restroke patients.

**Table 2:** sociodemographic characteristics of restroke patients (N=44)

Variable	Population study (n=44)
**Age(years)**	
15- 39	1(2.3%)
40-59	11(25%)
≥ 60	32(72.2%)
**Gender**	
Male	32(72.7%)
Female	12 (27.3%)
**Residence**	
Urban	30(68.2%)
Rural	14(31.8%)
**Marital status**	
Single	3(6.8%)
Married	33(75%)
Divorced	2(4.5%)
Widowed	6(13.6%)
**Level of education**	
None	32(68.2%)
Primary school	5 (11.4%)
Secondary school	3(6.8%)
University	4(9%)

**Vascular risk factors**: hypertension was the most vascular risk factors in 81.8% (n=36), followed by chronic alcohol consumption in 45.5 % (n=20) and overweight in 40.9% (n=18). Seventeen (38. 6 %) patients had three or more vascular risk factors. Twenty-five (69.4%) of hypertensive patients, had taken correctly their treatment. The frequency of hypertension was respectively 83.8% in first stroke patients and 71.4% in second stroke patients. The duration of hypertension was 5.5±4.33 years (ranges: 9 months and 20 years). Table 3 describes the distribution of restroke patients according to vascular risk factors.

**Table 3:** distribution of restroke patients according to vascular risk factors (N= 44)

Vascular risk factors	Study population (N=44)
Hypertension	36 (81.8%)
Alcool consomption	20(45.5%)
Tobacco use	13(29.5%)
Diabete Mellitus	2(4.5%)
Overweight	18 (40.9%)
Obesity	4(9.1%)

**Clinical characteristics of restroke**: at admission, six (13.6%) patients had coma and 4 (9.1%) were hypertensive. The most neurological deficit was motor deficit (100%), aphasia (84.1%; 37), hypoesthesia (14; 31.8%), and deglutition disorders (7; 15.9%). Recurrent stroke was ischemic in 36 (82%) cases and hemorrhagic in 8 (18%) cases. Brain MRI was performed in one patient. Electrocardiogram, echocardiography and cervical Doppler was performed in respectively 37 (84%) patients, 38 (86.4%) and 10 (22.7%). Blood investigation was performed in 38 (86.4%) patients. hyperglycemia (> 6 mmol/l) was found in 21 (52.5%) patients. The carotid plaque was found in 33 (75%) with tight stenosis in 2 patients. The most common cause of ischemic restroke was atherosclerosis in 23 (63.9%) patients, cardioembolic stroke in 12 (33.3%) patients and unknown cause in a patient (2.8%). All recurrent hemorrhagic stroke was hypertensive (100%).

**Previous stroke**: recent previous stroke was ischemic in 27 (61.4%) cases, hemorrhagic in 10 cases (22.72%) and unknown in 7 cases (15, 92%). The majority of the patients (70.6%) had their previous stroke >12 months before the admission. The etiology of previous stroke was unknown. The majority of the patients were treated with antiplatelet treatment (88.6%) when arriving at the hospital. Five (50%) of hemorrhagic patients were treated with antiagregant. Eighty-five percent of patients were treated with a lipid-lowering agent (85.4%) and 81.8% with hypertensive agents. in 39 (88.6%) patients. Poor compliance (<60%) was determined in patients taking antiagregant agents (43.6%) and statins (50%). Thirty (68.2%) patients had motor sequels and 24 (54.5%) had symptoms of depression after their first stroke. The cause of discontinuation was cost of drugs (27; 61.4%), intervention of a third party in the purchase of drugs (10; 22.7%), the use of traditional products (5; 11.4%). Table 4 describes the treatment of previous stroke.

**Table 4:** treatment of recent previous stroke (N=44)

Treatment	Frequency: n (%)	Compliance (%)
Antiagregant agents	39(88.6%)	17(43.6%)
Statins	38(85.4%)	19(50%)
Antihypertensive agents	36(81.8%)	25(69.4%)
Physiotherapy	31(70.5%)	19(61.3%)
Anticoagulant	2(4.5%)	100%

**Number of stroke recurrence**: first stroke was ischemic in 27 (61.4%) cases, hemorrhagic in 9 (20.4%) cases and unknown nature in 8 cases (18.2%). The mean interval time between initial and second stroke was 24 ± 24.34 months, ranges (3 months-10 years). The majority of patients (59.5%) had their stroke after 12 months. This time was 5.85 years between second stroke and third stroke. Table 5 gives the distribution of stroke subtype according to number of stroke. With the analysis of second stroke, recurrent stroke after intracerebral hemorrhage was hemorrhagic in 77.8% (7/9) and ischemic in 22.2% (2/9). Recurrent stroke after ischemic stroke was ischemic in 100% (24/24). Cerebral infarction after unknown nature were 100% (4/4).

**Table 5:** distribution of stroke subtype according to number of stroke

	Number of stroke		
**Stroke subtype**	1(n=44)	2(n=44)	3(n=7)
Ischemic	27(61.4%)	33(75 %)	6(85.7%)
Hemorrhagic	9(20.4%)	8(18.2 %)	1(14.3%)
Unknown	8(18.2%)	3(6.8%)	0

## Discussion

This observational study was the first kind in Burkina Faso to describe the frequency of stroke recurrence, the interval between initial stroke and recurrence, sociodemographic characteristic of patients with recurrent stroke and clinical characteristics of recurrent stroke. The recurrence stroke rate (16.5%) was similar than in a study in Cameron (14.5%) [11], in Germany (15%) [17], in Singapore (15.7%) [18] and in Egypt (12.9%) [19] but lower than in Turkey (26.9%) [20]. A lower frequency was observed in Ethiopia (8.8%) [13]. The difference between the studies is due to methodological features. According to number of stroke recurrence, 13.9% of patients had one recurrence and 2.6% had two recurrences. The same findings were observed by Stahmeyer in Germany [17] with respectively 12.1% and 2.1% of cases. The rate of recurrence was highest (62.50 %) following the first stroke in a Nigerian study [12].

The mean age of the patients with recurrent stroke was 66.5 ± 11.49 years was similar than in studies from Cameron (62 years) [11] and Saudi Arabia (67.70 ± 2 years) [21] but lower than in a study from Suisse, a western country (77 years) [22]. Stroke occurred in young age in Africa than in Europe due to the long expectancy in Western countries. There was a male predominance in our study (72.7%), in contrast with the study of (53%) [17]. In contrast with this study in which women were notably older than the men, men were older in our study (68.5 ± 11.93 versus 57.5 ± 8.97 years). The majority of the patients (70.6%) had their previous stroke > 12 months before the admission, in line with Leo (75%) [22]. This interval was 24 ± 24.34 months between first and second stroke, shorter than in the study of Zhu in China (58.42 months) [23]. In a study from Germany, the mean elapse of time between initial stroke event and recurrence was 697 days [17]. The majority of patients (59.5%) had their first restroke 12 months after first stroke. This may indicate that these patients were most vulnerable during the first year after their first stroke. This study confirms the important previous finding that the risk of stroke recurrence is highest in the immediate period after index stroke [24,25].

Hypertension was identified as a risk factor of stroke recurrence. Our study had found a high frequency of hypertension among recurrent stroke patients (91%), in line with the study of El-Gohary (90.2%) [21] and Yalcin (89%) [26], Kocaman (88%) [27], Morsy (100%) [28] and Cámara (80%) [29] but lower frequency was observed in the study of Leo (75%) [22] and Stahmeyer (69%) [17]. Some others authors in Scotland [25] and Thailand [30] demonstrated that hypertension was not identified as a risk factor of stroke recurrence. Another factor that is most commonly associated with stroke recurrence is diabetes [30-32]. This figure was not observed in our study (4.5%). The majority of recurrent stroke were ischemic (82%), in the same proportion than in the study of Stahmeyer (81%) [17] and El-Gohary [21] in Saudi Arabia (83.93%). Previous stroke was ischemic in 61.4%, in lower frequency than in the study of Leo (90%). The most treatment after previous stroke was antiagregant agent (88.6%), statins (85.4%) and antihypertensive agent (81.8%). Discontinuation of aspirin agents is a risk factor for stroke; however, it is underestimated by most physicians [33]. In our study, discontinuation of aspirin agents was observed in 56.4%, in similar proportion than in the study of Negm (51%) [34]. Antithrombotic therapy following ICH was observed in 50% of previous hemorrhagic stroke. The other drug discontinuation was statins (50%) and antihypertensive agents (30.6%). Recurrent stroke after intracerebral hemorrhage was hemorrhagic in 77.8% and ischemic in 22.2%, in line with Bailey who affirms that recurrent brain hemorrhage is more frequent than ischemic stroke after intracranial hemorrhage [35]. The frequency of recurrent hemorrhagic stroke after hemorrhagic stroke was higher than in literature (0-24%) [36]. In our study, all the patients with initial ischemic stroke had recurrent ischemic (100%). This frequency was largely higher than in literature (18%) [4,9,27,37-39]. The higher frequency of recurrent hemorrhagic stroke could be explained by the shorter of our sample.

**Study limitations**: this observational study had several limitations. We did do statistical analysis about the risk factors of recurrent stroke in African context. The size of the study population with recurrent stroke (ischemic or hemorrhagic) was small. The records of some patients regarding the etiology and detailed treatment at the first episode of stroke were missing.

## Conclusion

Stroke recurrence is common in our context. Hypertension was the most common vascular risk factor in recurrent stroke. Poor compliance was determined in patients taking antiagregant agents and statins in previous stroke.

### What is known about this topic

- Restroke is a major contributor to disability and mortality in patients with stroke;
- The recurrence rate of restroke is variable according to the duration of follow-up period ischemic;
- Ischemic restroke is frequent than hemorrhagic restrike.

### What this study adds

- This study can be add to African literature because studies on restroke are very rare;
- This study shows the most common vascular risk factors in restroke;
- This study shows the frequency of first, second and third stroke according to number of stroke.
